# Characteristics of Graphene Oxide Films Reduced by Using an Atmospheric Plasma System

**DOI:** 10.3390/nano8100802

**Published:** 2018-10-08

**Authors:** Chii-Rong Yang, Shih-Feng Tseng, Yu-Ting Chen

**Affiliations:** 1 Department of Mechatronic Engineering, National Taiwan Normal University, Taipei 10610, Taiwan; ycr@ntnu.edu.tw (C.-R.Y.); c010321@hotmail.com (Y.-T.C.); 2 Department of Mechanical Engineering, National Taipei University of Technology, Taipei 10608, Taiwan

**Keywords:** oxygen functional group, plasma irradiation, GO and rGO films, sheet resistance, carbon-to-oxygen ratio

## Abstract

The chemical oxidation method can be used to mass-produce graphene oxides (GOs) from highly oriented pyrolytic graphite. However, numerous oxygen-containing functional groups (hydroxyl, epoxy, carbonyl, etc.) exist in typical GO surfaces, resulting in serious electrical losses. Hence, GO must be processed into reduced graphene oxide (rGO) by the removal of most of the oxygen-containing functional groups. This research concentrates on the reduction efficiency of GO films that are manufactured using atmospheric-pressure and continuous plasma irradiation. Before and after sessions of plasma irradiation with various irradiation times, shelters, and working distances, the surface, physical, and electrical characteristics of homemade GO and rGO films are measured and analyzed. Experimental results showed that the sheet resistance values of rGO films with silicon or quartz shelters were markedly lower than those of GO films because the rGO films were mostly deprived of oxygen-containing functional groups. The lowest sheet resistance value and the largest carbon-to-oxygen ratio of typical rGO films were approximately 90 Ω/sq and 1.522, respectively. The intensity of the C–O bond peak in typical rGO films was significantly lower than that in GO films. Moreover, the intensity of the C–C bond peak in typical rGO films was considerably higher than that in GO films.

## 1. Introduction

Graphene is a single layer of carbon atoms with a two-dimensional hexagonal lattice structure. A graphene film is an ultrathin carbon film, approximately 0.34 nm in thickness. This material was first discovered by Geim and Novoselov, who used adhesive tape to repeatedly cleave highly oriented pyrolytic graphite (HOPG) [1,2]. Due to its excellent optical, electrical, mechanical, physical, and chemical properties, numerous researchers and scholars have developed promising applications of graphene for products such as diaplay panels [3], solar cells [4], electronic circuit boards [5], corrosion prevention [6], light-emitting diodes (LEDs) [7], supercapacitors [8], senesing devices [9], inspection chips [10], and thermal heating chips [11]. In recent decades, many methods have been proposed for fabricating graphene materials, including micromechanical cleavage of HOPG [12], epitaxial growth method [13], chemical vapor deposition (CVD) [3], electrochemical exfoliation of HOPG [14], light exfoliation [15], chemical exfoliation [16], and reduction of graphene oxide (GO) [17]. The technology for reducing GO to graphene is a mass production method that can manufacture graphene materials by the ton. Consequently, numerous methods for thermal reduction had been proposed. Schniepp et al. [18] used thermal treatment reduction to fabricate reduced graphene oxide (rGO) samples. First, GO samples were prepared according to the Staudenmaier method. These GO samples were placed at the end of a quartz tube, and then the quartz tube was inserted into a furnace preheated to 300 °C, 500 °C, 750 °C, or 1050 °C to exfoliate the graphite oxide. After the X-ray photoelectron spectroscopy (XPS) analysis, an oxygen/carbon atomic ratio (O/C) of GO samples reduced from 38% down to 10% between the thermal treatment temperature of 300 °C and 1050 °C. Ramesha and Sampath [19] utilized electrochemical reduction to produce rGO from oriented GO films. This method can be used to reduce GO by applying a DC bias voltage from 0 to −1 V. Their initial GO samples had a G band at 1610 cm^−1^. When they measured their rGO with Raman spectroscopy, the G band shifted to 1585 cm^−1^. Stankovich et al. [20] applied chemical reduction to reduce exfoliated graphite oxide sheets in water with hydrazine hydrate, and thus produced rGO sheets. The folded regions of the rGO sheets were observed using a high-resolution scanning electron microscope (SEM); the folded regions had average widths of approximately 2 nm. In the analyzed results of C1s XPS spectra, the peak intensities of oxygen functionalities in rGO sheets were much smaller than those in GO sheets. Williams et al. [21] obtained rGO with a UV-assisted reduction technique, irradiating GO in TiO_2_ suspensions. After this reduction process, the color of the GO suspension changed from brown to black. Zhu et al. [22] reported a microwave irradiation method to rapidly and efficiently achieve the exfoliation and reduction of graphite oxide. Some reduced graphite oxide powders were rapidly obtained in a commercial microwave oven within 1 min. The specific capacitance value of an ultracapacitor cell made by using such reduced graphite oxide powders as the electrode material was 191 F/g. Kasischke et al. [23] reported on the simultaneous reduction and periodic patterning of GOs by using a femtosecond laser processing system. The sheet resistance of GOs irradiated with 10^4^ pulses per spot and laser fluences ranging from 11 mJ/cm^2^ to 34 mJ/cm^2^ ranked from 0.86 ± 0.01 kΩ/sq to 2.8 ± 0.01 kΩ/sq, respectively. Furthermore, the rGO surface produced laser-induced periodic surface structures (LIPSS) when the laser fluence ranged from 22.5 mJ/cm^2^ to 48 mJ/cm^2^. Baraket et al. [24] used electron beams to generate CH_4_ plasmas and then to reduce GO flakes. The XPS spectra revealed that the C1s peak of rGO flakes with plasma irradiation for 60 s and 90 s was obviously higher than that of GO flakes. The ratio of the G to D peak intensities (*I_D_*/*I_G_*) of GO and rGO flakes varied between 0.9 and 1 and between 0.7 and 1, respectively. Dey et al. [25] proposed an approach to print GO films from highly acidic suspensions combined with in situ reduction by an atmospheric pressure plasma system with low temperature plasma of a He/H_2_ gas mixture. The measured results of XPS and near-edge X-ray absorption fine structure (NEXAFS) showed that the carboxylic acid functionalities were reduced to phenol groups during the deposition step. Moreover, the oxygen content at the surface was reduced with extended exposure to the plasma jet. This method had a strong potential to print conducting patterns of rGO for flexible electronic devices and energy storage devices.

To meet requirements for the low cost, simple process, easy control reduction area, and mass production for rGO films, this study aimed to develop a novel thermal reduction method for GO films using an atmospheric-pressure plasma system. Because this process can produce rGO films under atmospheric pressure and without vacuum, this process could reduce manufacturing costs, could be easily operated, and could streamline the maintenance of associated manufacturing equipment. The variables of atmospheric plasma reduction in this study were various plasma irradiation times, shelters, and working distances. Before and after atmospheric plasma irradiation, the surface morphologies, Raman spectra, and O/C ratios of GO and rGO films were observed and analyzed using an SEM, a Raman spectroscope, and an XPS, respectively. Furthermore, the sheet resistance values of GO and rGO films were measured using a four-point probe instrument.

## 2. Experimental Details

**Figure 1:**
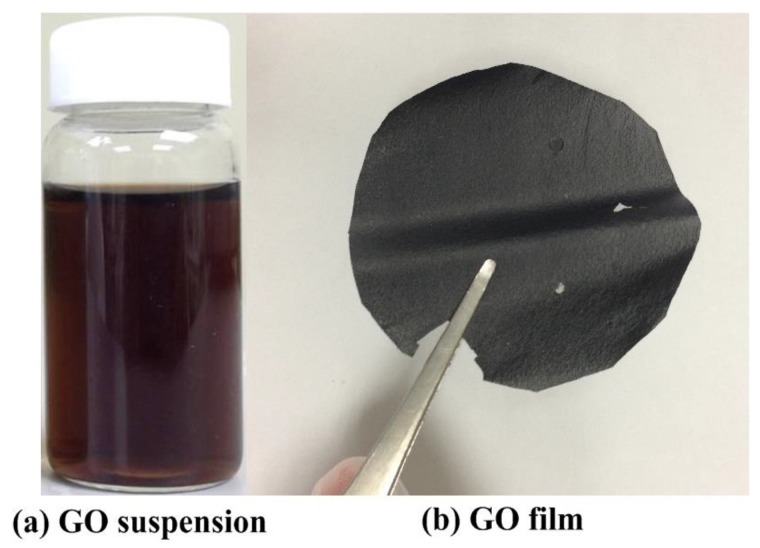
Digital picture of graphene oxide (GO) suspensions (**a**) and films (**b**) peeled off from the Si substrate.

**Figure 2:**
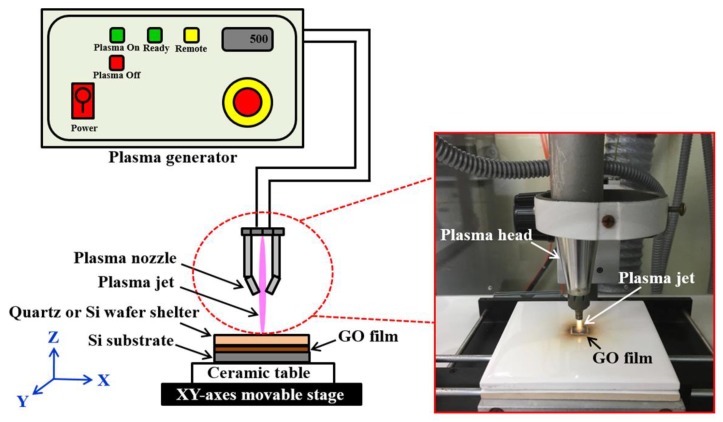
Schematic of the atmospheric-pressure plasma system with a digital image of the plasma head.

The homemade GO films were fabricated from HOPG sheets using peroxide, deep-oxide, and post-treatment steps. Through an improved version of Hummers’ method, GO suspensions with a concentration of 1 mg/mL were obtained. The experimental details of the produced GO suspensions were described in our previously published study [26]. The GO suspensions were coated onto silicon (Si) wafer surfaces with a drop-casting method. Moreover, the GO films were dried at room temperature for 48 h. Figure 1 shows digital pictures of GO suspensions (a) and films (b). The typical thickness of GO films is about 150 μm. The dimension of GO specimens for atmospheric plasma reduction is 10 mm × 10 mm. Figure 2 schematically illustrates the atmospheric-pressure plasma system with a digital picture of a plasma head. The atmospheric-pressure plasma system (Diener electronic series plasma beam, Germany) mainly consisted of a power supply, a plasma generator, a plasma head, and a ceramic table on an XY-axes movable stage with a ball-screw mechanism. The plasma head was set perpendicularly to the surface of the ceramic table. Each dry GO film coated on the silicon wafer was placed between the ceramic table and a shelter. To avoid blowing off the GO films during the atmospheric plasma reduction process, double-sided polished silicon wafers or quartz glass panels served as shelters. The hole diameter of the plasma nozzle at the end of the plasma head was approximately 2 mm. In the reduction of GO films, the plasma output power (*P*) was fixed at 300 W. The thermal reduction parameters included the plasma irradiation times (30, 60, 90, and 120 min), the shelter types (quartz glass or silicon), and the working distances (*D*) between the tip of the plasma nozzle and the specimen surface (9 mm and 18 mm). Clean, dry, pressurized air was selected as the processing gas for the plasma reduction process. The working air pressure was set at 5 kg/cm^2^. Plasma could be produced by heating neutral gas particles to become ionised. Moreover, the plasma could be used to remove oxide layers on material surfaces. Table 1 presents a summary of the plasma irradiation parameters used to reduce GO films on the Si substrates.

An SEM (Model: JSM-6360LV, JEOL Inc., Tokyo, Japan), a Raman spectroscope (Model: NRS-4100, JASCO Inc., Tokyo, Japan), and an XPS (Model: Theta Probe, Thermo Fisher Scientific Inc., East Grinstead, UK) were used to measure and analyze certain physical properties of the GO specimens before and after plasma irradiation. The surface morphologies of GO and rGO films were observed by using the SEM. The Raman spectra were obtained by using an excitation laser with a wavelength of 532 nm at room temperature. Moreover, the measured laser power was limited to 2 mW to avoid laser-induced heating on the specimen surfaces. The O/C ratios of GO and rGO specimens were obtained by using the XPS with X-ray spot size ranging from 15 to 400 µm. This XPS instrument had an automatic motorized stage; furthermore, the maximum field of view was 70 × 70 mm^2^. A four-point probe (Model: 5601Y, Chitai Electronic Inc., Taipei, Taiwan) was employed to measure the electrical conductivity of rGO specimens.

## 3. Results and Discussion

### 3.1. Surface Morphologies of GO Films before and after Plasma Reduction

**Figure 3:**
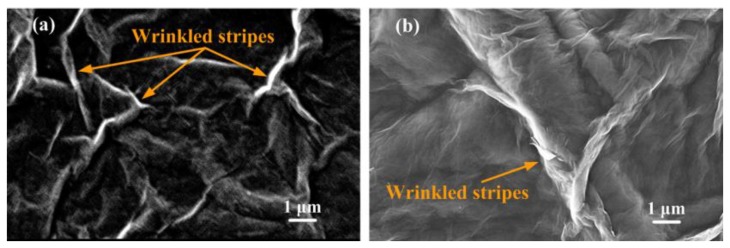
Typical SEM images of the GO film (**a**) and reduced graphene oxide (rGO) film (**b**) coated on the Si substrate.

Some GO sheets were dispersed in 100 mL of deionized water to produce GO suspensions. Before the drop-casting of GO suspensions at a concentration of 1 mg/mL, the SiO_2_/Si substrates were cleaned through sonication in acetone (CH_3_CO), deionized water, and isopropanol (C_3_H_8_O) sequentially to remove the olein that adhered to the substrate surfaces. To increase the hydrophilicity of the SiO_2_/Si substrates, clean and dry pressurized air was converted to plasma and used to clean each substrate surface for 30 s. GO suspensions were perpendicularly drop-cast on the SiO_2_/Si wafer substrates, producing GO films on the substrates. Then, GO films were dried at room temperature for 48 h. Because GO films do not offer sufficient electrical conductivity, each GO film was coated with a gold thin film that served as a conductive layer for surface morphology measurements using the SEM. Figure 3 presents typical SEM images of a GO film (a) and an rGO film (b) coated on Si substrates. The rGO film was reduced with a working distance of 9 mm and a plasma irradiation time of 120 min and covered with a quartz shelter. Both images were measured at 10,000× magnification. The GO film surface exhibited markedly wrinkled microstripes and nanostripes because the violent oxidizer destroyed the ordered structures of HOPG sheets during the deep-oxide process; therefore, the bond angle of carbon–oxygen bonds was altered, as shown in Figure 3a. After the plasma reduction, the wrinkled stripes on the rGO film surface were markedly decreased, as shown in Figure 3b. Moreover, the surface morphology of rGO films became more bouffant due to the intense plasma-jet interaction.

### 3.2. Raman Spectra Analysis

**Figure 4:**
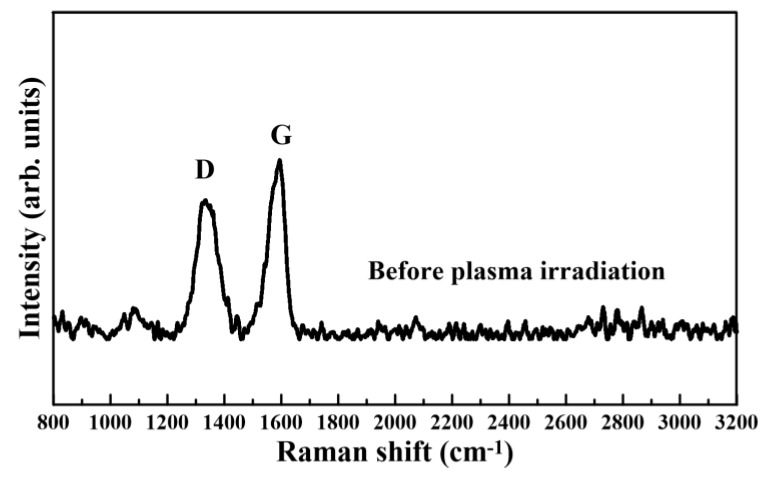
Raman spectrum measured on GO films before plasma irradiation.

**Figure 5:**
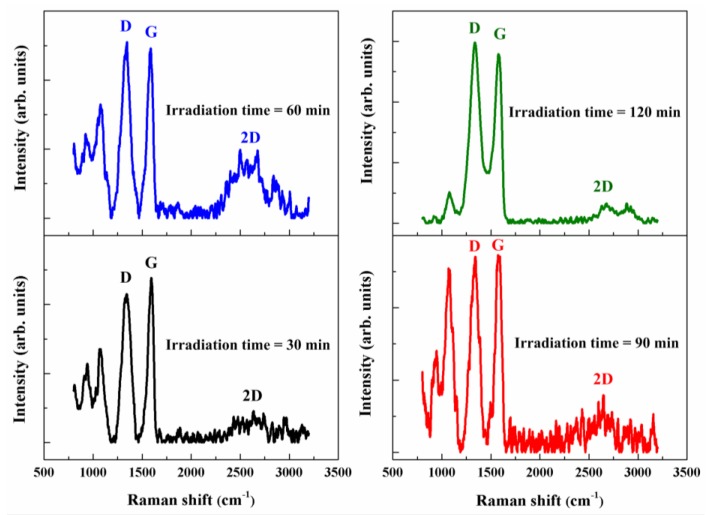
Raman spectra measured on rGO films with different irradiation times of atmospheric-pressure plasma without any shelter.

Figure 4 and Figure 5 present the Raman spectra measured on GO films before and after plasma irradiation without any shelter: the Raman shifts range from 800 to 3200 cm^−1^. Before the plasma reduction, the measured Raman spectra of GO films only exhibited D and G bands, as depicted in Figure 4. The intensity of the D band was lower than that of the G band. The measured Raman spectra of GO films after the plasma irradiation had three characteristic peaks for the D, G, and 2D bands centered at 1338, 1580, and 2679 cm^−1^, respectively, as graphed in Figure 5. The D, G, and 2D bands represented the material defects, the in-plane vibration of the sp^2^ carbon atoms, and the stacking order, respectively. The plasma output power and working distance were respectively fixed at 300 W and 18 mm without any shelter. Moreover, the variable parameters were the irradiation times of atmospheric-pressure plasma (30, 60, 90, and 120 min). The measured Raman spectra of all rGO films graphed in Figure 5 revealed that the obvious appearance of the 2D peak corresponded to graphene formation because of the plasma-induced reduction reaction. Notably, the 2D peak of rGO films with a plasma irradiation time of 60 min was higher than those of rGO films with different plasma irradiation times.

According to the measured results displayed in Figure 5, the intensity of the D band of rGO films with a plasma irradiation time of 120 min was significantly lower than that of other films. This occurred because long plasma irradiation sessions produced large defects that broke the rGO films. The ratios of D/G relative intensity (*I_D_*/*I_G_*) and 2D/G relative intensity (*I_2D_*/*I_G_*) were normally used to quantify defects in graphene materials and to qualify the number of stacked graphene layers, respectively. The *I_D_*/*I_G_* and *I_2D_*/*I_G_* ratios of GO and rGO films without any shelter for different plasma irradiation times are summarized in Table 2. The average *I_D_*/*I_G_* ratio of GO films was 0.77. The *I_D_*/*I_G_* ratios of rGO films were 0.89, 1.08, 0.97, and 1.09 when the plasma irradiation times were 30, 60, 90, and 120 min, respectively. The *I_D_*/*I_G_* ratios of rGO films were markedly larger than those of GO films because the plasma irradiation produced some material defects. The *I_2D_*/*I_G_* ratios of rGO films were 0.08, 0.23, 0.19, and 0.05 when the plasma irradiation times were 30, 60, 90, and 120 min, respectively. The measured results of Raman spectra proved that the *I_2D_*/*I_G_* ratios of rGO films increased with increasing plasma irradiation times ranging from 30 to 60 min. Nevertheless, the peak intensity of the 2D band declined as the plasma irradiation time increased to 120 min. This phenomenon was attributed to the prolonged interaction forces of plasma jets on the GO film surface, which blew out the rGO films and reduced the plasma reduction ability. Nguyen et al. reported that *I_2D_*/*I_G_* ratios of 2–3, 1–2 and less than 1 represented monolayer, bilayer, and multilayer graphene, respectively [27]. In this study, the *I_2D_*/*I_G_* ratios of rGO films without any shelter were less than 1; thus, it was estimated that the products had multilayer graphene structures.

**Figure 6:**
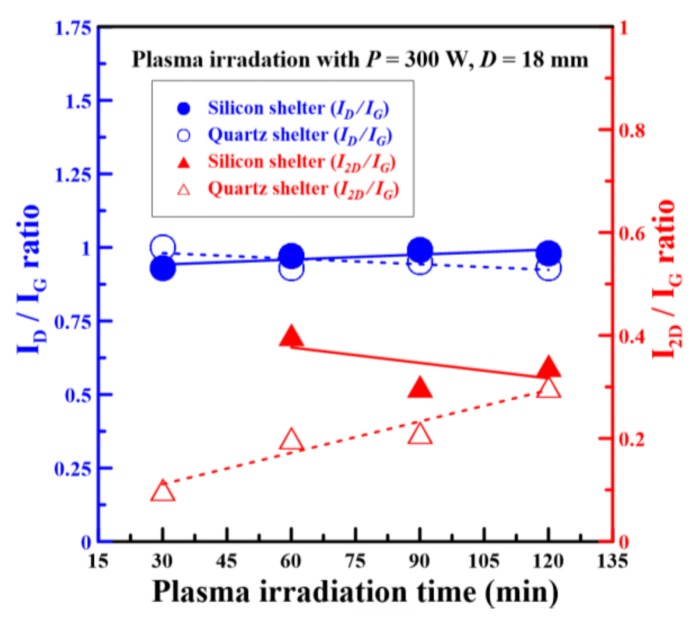
Summary of the *I_D_/I_G_* and *I_2D_/I_G_* ratios of rGO films versus different plasma irradiation times with silicon and quartz shelters.

To ameliorate the GO film blow-off problem on the Si substrate during the aforementioned plasma reduction process, double-sided polished shelters made of silicon and quartz were individually selected as shelters for the GO film surfaces. Figure 6 shows the *I_D_/I_G_* and *I_2D_/I_G_* ratios of rGO films versus different plasma irradiation times with silicon and quartz shelters. The *I_D_/I_G_* ratios of rGO films with silicon and quartz shelters were 0.93, 0.97, 0.99, and 0.98 and 1, 0.93, 0.95, and 0.93 when the plasma irradiation times were 30, 60, 90, and 120 min, respectively. The *I_D_/I_G_* ratios of rGO films sheltered by silicon were increased slightly by increasing the plasma irradiation times, as plotted by the solid blue line in Figure 6. However, the *I_D_/I_G_* ratios of rGO films sheltered by quartz were decreased slightly by increasing plasma irradiation times, as plotted by the solid red line in Figure 6. This implied that the material defects of rGO films sheltered by silicon were slightly larger than those of rGO films sheltered by quartz as plasma irradiation times increased. After the plasma reduction of silicon-sheltered GO films for 30 min, the 2D peak of Raman spectra disappeared because the silicon was an opaque material through which the plasma jet could not directly interact with GO films. Hence, the plasma irradiation required a long time to accumulate heat and to reduce the GO films. By increasing the plasma irradiation time from 60 to 120 min, the *I_2D_/I_G_* ratios of silicon-sheltered rGO films were 0.4 and 0.34, respectively. Conversely, the *I_2D_/I_G_* ratios of quartz-sheltered rGO films were 0.1, 0.2, 0.21, and 0.3 when the plasma irradiation times were 30, 60, 90, and 120 min, respectively. The *I_2D_/I_G_* ratios of quartz-sheltered rGO films were significantly increased by increasing plasma irradiation times, as plotted by the dashed red line in Figure 6. The *I_D_/I_G_* and *I_2D_/I_G_* ratios of rGO films with silicon and quartz shelters for different plasma irradiation times are summarized in Table 3. All *I_2D_/I_G_* ratios of sheltered rGO films were less than 1, and therefore all those films were estimated to have multilayer graphene structures. In the context of all films with atmospheric plasma reductions, the silicon-sheltered rGO film with a plasma irradiation time of 60 min had the highest *I_2D_/I_G_* ratio of 0.4.

### 3.3. Electrical Resistance Analysis

**Figure 7:**
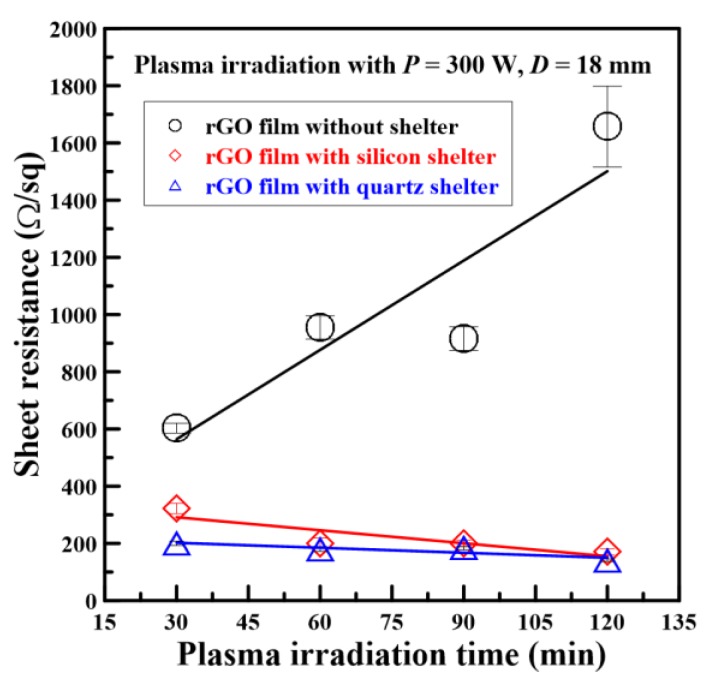
Sheet resistance values of rGO films with and without shelters versus different plasma irradiation times.

Before and after the plasma irradiation sessions (which had various durations), the electrical resistance values of GO and rGO films with and without shelters were measured using the four-point probe. The measured results showed that the sheet resistance values of GO films were 280 MΩ/sq. During the plasma reduction process, the plasma output power and working distance were fixed at 300 W and 18 mm, respectively. When the plasma irradiation times were 30, 60, 90, and 120 min, the sheet resistance values of unsheltered, silicon-sheltered, and quartz-sheltered rGO films were 603, 955, 916, and 1657 Ω/sq; 322, 200, 201, and 170 Ω/sq; and 200, 180, 183, and 141 Ω/sq, respectively. The sheet resistance values of rGO films with and without shelters versus different plasma irradiation times are depicted in Figure 7. After plasma irradiation, all sheet resistance values of rGO films were considerably lower than those of the GO films. According to Gao et al. [28], four types of oxygen functionalities subsisted in GO, namely epoxide (–O–), hydroxyl (–OH), carbonyl (–C=O), and carboxyl (–COOH). The epoxides inside a sample of GO could be removed at reaction temperatures of 100 to 150 °C. In this study, the plasma-induced thermal temperature was approximately 400 °C; this temperature could easily remove epoxide groups from GO films and increase electrical conductivity. However, the sheet resistance values of rGO films without any shelter gradually increased as the plasma irradiation time increased; electrons could not move easily because the film surfaces were crumbled by plasma jets. To improve the products, shelters were used to cover the GO film surfaces. Therefore, the sheet resistance values of sheltered rGO films were markedly lower than those of unsheltered films. In particular, the sheet resistance values of quartz-sheltered rGO films were obviously lower than those of silicon-sheltered films for each plasma irradiation time.

**Figure 8:**
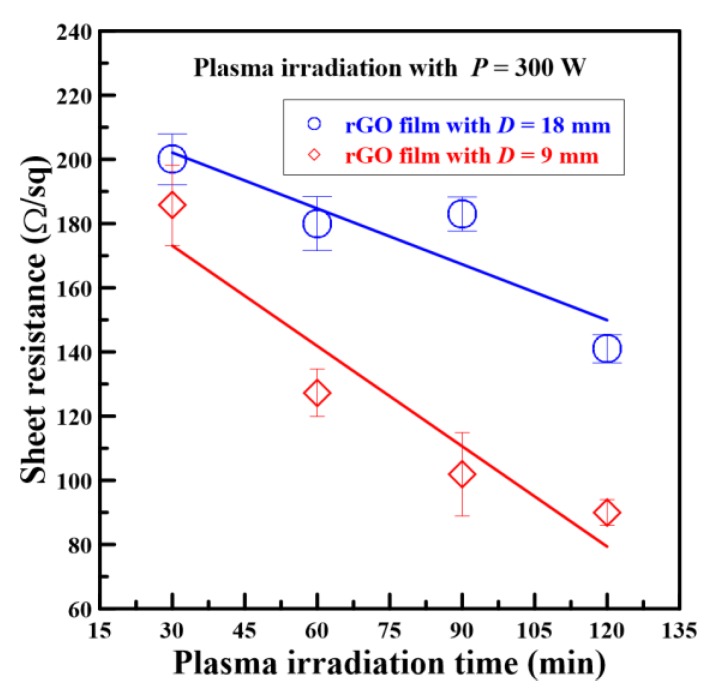
Sheet resistance values of rGO films sheltered by quartz versus different plasma irradiation times and working distances.

Because the quartz-sheltered rGO films had lower sheet resistance values, to investigate the variety of sheet resistance values, the working distance was changed from 18 mm to 9 mm so that the plasma jets transferred considerable heat onto the film surfaces. Figure 8 shows the sheet resistance values of quartz-sheltered rGO films versus different plasma irradiation times and working distances. The sheet resistance values of quartz-sheltered rGO films were 185.7, 127.3, 101.9, and 90 Ω/sq when the plasma irradiation times were 30, 60, 90, and 120 min. The measured results revealed that the sheet resistance value decreased as the plasma irradiation time increased. Moreover, all sheet resistance values for rGO films fabricated at a working distance of 9 mm were substantially lower than those of ones fabricated at a working distance of 18 mm.

### 3.4. XPS Analysis

**Figure 9:**
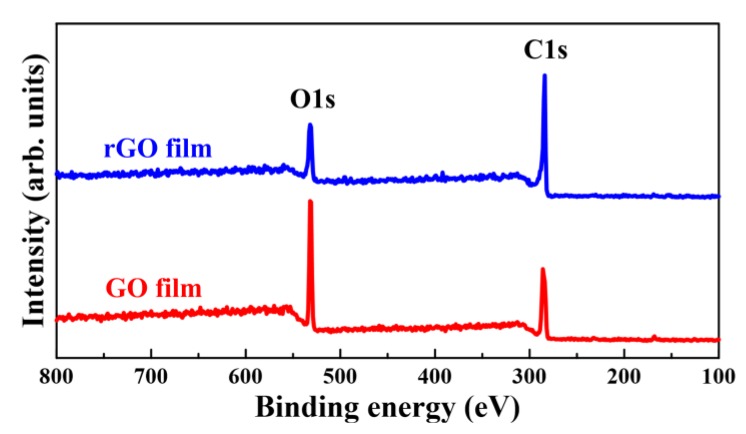
XPS analysis of GO and typical rGO films sheltered by quartz with a working distance of 9 mm and plasma irradiation time of 120 min.

To investigate the degree of reduction of rGO films, XPS was used to analyze the chemical composition before and after plasma reduction sessions with different irradiation times. Figure 9 shows the C1s (286.7 eV peak) and O1s (532.6 eV peak) XPS spectra of quartz-sheltered GO and rGO films, with a working distance of 9 mm and a plasma irradiation time of 120 min. The carbon and oxygen contents and the carbon-to-oxygen (C/O) ratio of the GO films were 24.73%, 75.27%, and 0.329, respectively. The red line of Figure 9 indicates that the O1s peak was distinctly higher than the C1s peak for GO films. The carbon and oxygen contents and the C/O ratio of quartz-sheltered rGO films with a working distance of 9 mm and a plasma irradiation time of 120 min were 60.35%, 39.65%, and 1.522, respectively. The blue line of Figure 9 indicates that the C1s peak was distinctly higher than the O1s peak for rGO films. The oxygen and carbon contents and C/O ratios of GO and rGO films with a plasma irradiation time of 120 min with different shelters and working distances are summarized in Table 4. When the applied working distance was changed from 18 to 9 mm, the oxygen content of quartz-sheltered rGO films changed from 43.53% to 39.65%. The oxygen content declined from 75.27% in the initial GO films to 39.65% in the quartz-sheltered rGO films, with a working distance of 9 mm and a plasma irradiation time of 120 min. This indicated that a plurality of oxygen functionalities was removed and efficient reduction occurred after the plasma irradiation on GO films. The C/O ratio of GO films was 0.329. The C/O ratio of silicon-sheltered rGO films with a working distance of 18 mm and a plasma irradiation time of 120 min was 1.33. Moreover, the C/O ratios of quartz-sheltered rGO films, with working distances of 9 and 18 mm and with a plasma irradiation time of 120 min were 1.297 and 1.522, respectively. The C/O ratios of all rGO films were larger than those of GO films.

**Figure 10:**
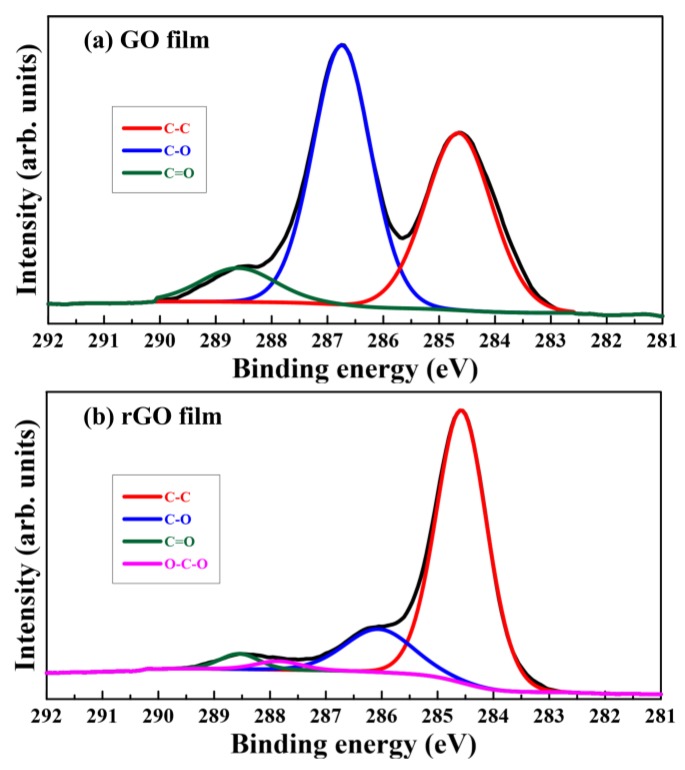
C1s XPS spectra of GO (**a**) and typical rGO (**b**) films sheltered by quartz with a working distance of 9 mm and plasma irradiation time of 120 min.

To resolve the issues related to these bond components, C1s XPS spectra of GO films and typical quartz-sheltered rGO films with a working distance of 9 mm and plasma irradiation time of 120 min were deconvoluted in Figure 10a,b, respectively. Figure 10a shows three types of covalently bonded carbon existing in GO films as C–C (284.6 eV), C–O (286.7 eV), and C=O (288.6 eV), respectively. Furthermore, the four types of covalently bonded carbon structures existing in typical rGO films were C–C (284.6 eV), C–O (286.7 eV), C=O (288.6 eV), and O–C–O (287.8 eV), as presented in Figure 10b. A new peak of conjugated O–C–O structures at 287.8 eV appeared in the C1s spectra of typical rGO films. This was attributed to the plasma reduction process with clean dry air, which produced a new bond. Because the GO films had numerous oxygen-containing functional groups, the intensity of the C–O bond peak was clearly higher than that of the C–C bond peak. However, the intensity of the C–O bond peak was markedly lower than that of the C–C bond peak after plasma reduction. This implied that oxygen-containing functional groups of typical rGO films decreased as carbon atoms increased. A comparison of Figure 10a,b indicates that the intensities of the C–O bond peaks in typical rGO films were significantly lower than those in GO films, and the intensities of the C–C bond peaks in typical rGO films were markedly higher than those in GO films.

## 4. Conclusions

The proposed atmospheric-pressure plasma system can reduce GO films coated on Si substrates. After plasma irradiation, the measured Raman spectra of all rGO films exhibited obvious 2D peaks. A typical unsheltered rGO film reduced with a plasma output power of 300 W, a plasma irradiation time of 60 min, and a working distance of 18 mm had the largest *I_2D_*/*I_G_* ratio of 0.23. Because oxygen-containing functional groups of typical rGO films mostly decreased, the sheet resistance values of rGO films with silicon or quartz shelters were obviously lower than those of GO films. A typical quartz-sheltered rGO film fabricated with a plasma output power of 300 W, a plasma irradiation time of 120 min, and a working distance of 9 mm had the lowest sheet resistance value of approximately 90 Ω and the largest C/O ratio of 1.522. In C1s XPS spectra, the intensities of the C–O bond peaks in typical rGO films were significantly lower than those in GO films. Moreover, the intensities of the C-C bond peaks in typical rGO films were considerably higher than those in GO films. Plasma reduction techniques of GO films has been successfully achieved in this study. In the future, this approach can be used in high-power plasma combined with an XY-axis motorized stage or multi-head plasma irradiation to achieve the mass reduction of large-scale GO films or GO powders. Moreover, the rGO films can be widely used in heat-conductive, thermoelectric, and electrically conductive layers of optoelectronic and semiconductor applications. 

## Figures and Tables

**Table 1:** Summary of the plasma irradiation parameters used to reduce GO films on the Si substrate.

Plasma Irradiation Parameters	Values or Methods			
Plasma output power (W)	300			
Plasma irradiation time (min)	30	60	90	120
Working distance (mm)	9		18	
Shelter meterial	Quartz		Si wafer	
Processing gas	Dry pressured air			
Air pressure (kg/cm^2^)	5			

**Table 2:** Summary of the *I_D_/I_G_* and *I_2D_/I_G_* ratios of GO and rGO films without any shelter for different plasma irradiation times.

Film Types	GO	rGO			
Plasma irradiation time (min)	-	30	60	90	120
*I_D_/I_G_*	0.77	0.89	1.08	0.97	1.09
*I_2D_/I_G_*	-	0.08	0.23	0.19	0.05

**Table 3:** Summary of the *I_D_/I_G_* and *I_2D_/I_G_* ratios of rGO films with silicon and quartz shelters for different plasma irradiation times.

Shelter Types	Silicon				Quartz			
Plasma irradiation time (min)	30	60	90	120	30	60	90	120
*I_D_*/*I_G_*	0.93	0.97	0.99	0.98	1	0.93	0.95	0.93
*I_2D_*/*I_G_*	×	0.4	0.3	0.34	0.1	0.2	0.21	0.3

**Table 4:** Summary of the oxygen and carbon contents and C/O ratios of GO and typical rGO films with the plasma irradiation time of 120 min.

Film Types	GO	rGO		
Shelter types	-	Silicon	Quartz	
Working distance (mm)	-	18	18	9
Oxygen content (%)	75.27	42.91	43.53	39.65
Carbon content (%)	24.73	57.09	56.47	60.35
C/O ratio	0.329	1.33	1.297	1.522

## References

[1] Novoselov K.S., Geim A.K., Morozov S.V., Jiang D., Zhang Y., Dubonos S.V., Grigorieva I.V., Firsov A.A. (2004). Electric field effect in atomically thin carbon films. Science.

[2] Geim A.K. (2009). Graphene: status and prospects. Science.

[3] Bae S., Kim H., Lee Y., Xu X., Park J.S., Zheng Y., Balakrishnan J., Lei T., Kim H.R., Song Y.I. (2010). Roll-to-roll production of 30-inch graphene films for transparent electrodes. Nat. Nanotechnol..

[4] Wang H., Sun K., Tao F., Stacchiola D.J., Hu Y.H. (2013). 3D honeycomb-like structured graphene and its high efficiency as a counter-electrode catalyst for dye-sensitized solar cells. Angew. Chem. Int. Ed..

[5] Hyun W.J., Park O.O., Chin B.D. (2013). Foldable graphene electronic circuits based on paper substrates. Adv. Mater..

[6] Prasai D., Tuberquia J.C., Harl R.R., Jennings G.K., Bolotin K.I. (2012). Graphene: Corrosion-Inhibiting Coating. ACS Nano.

[7] Cho E.C., Huang J.H., Li C.P., Chang-Jian C.W., Lee K.C., Hsiao Y.S., Huang J.H. (2016). Graphene-based thermoplastic composites and their application for LED thermal management. Carbon.

[8] Ke Q., Wang J. (2016). Graphene-based materials for supercapacitor electrodes—A review. J. Materiomics.

[9] Dunst K., Author Vitae Jurków D., Author Vitae Jasiński P. (2016). Laser patterned platform with PEDOT-graphene composite film for NO_2_ sensing. Sens. Actuators B Chem..

[10] Tseng S.F., Hsiao W.T., Cheng P.Y., Chung C.K., Lin Y.S., Chien S.C., Huang W.Y. (2016). Graphene-based chips fabricated by ultraviolet laser patterning for an electrochemical impedance spectroscopy. Actuators B Chem..

[11] Tseng S.F. (2018). Picosecond laser micropatterning of graphene films for rapid heating chips. Appl. Surf. Sci..

[12] Singh V., Joung D., Zhai L., Das S., Khondaker S.I., Seal S. (2011). Graphene based materials: Past, present and future. Prog. Mater. Sci..

[13] Ouerghi A., Ridene M., Mathieu C., Gogneau N., Belkhou R. (2013). From nanographene to monolayer graphene on 6H-SiC (0001) substrate. Appl. Phys. Lett..

[14] Su C.Y., Lu A.Y., Xu Y., Chen F.R., Khlobystov A.N., Li L.J. (2011). High-quality thin graphene films from fast electrochemical exfoliation. ACS Nano.

[15] Kim S.R., Parvez M.K., Chhowalla M. (2009). UV-reduction of graphene oxide and its application as an interfacial layer to reduce the back-transport reactions in dye-sensitized solar cells. Chem. Phys. Lett..

[16] Ciesielski A., Samori P. (2014). Graphene via sonication assisted liquid-phase exfoliation. Chem. Soc. Rev..

[17] Buglione L., Chng E.L.K., Ambrosi A., Sofer Z., Pumera M. (2012). Graphene materials preparation methods have dramatic influence upon their capacitance. Electrochem. Commun..

[18] Schniepp H.C., Li J.L., McAllister M.J., Sai H., Herrera-Alonso M., Adamson D.H., Prud’homme R.K., Car R., Saville D.A., Aksay I.A. (2006). Functionalized single graphene sheets derived from splitting graphite oxide. J. Phys. Chem. B.

[19] Ramesha G.K., Sampath S. (2009). Electrochemical reduction of oriented graphene oxide films: an in situ raman spectroelectrochemical study. J. Phys. Chem. C.

[20] Stankovich S., Dikin D.A., Piner R.D., Kohlhaas K.A., Kleinhammes A., Jia Y., Wu Y., Nguyen S.B.T., Ruoff R.S. (2007). Synthesis of graphene-based nanosheets via chemical reduction of exfoliated graphite oxide. Carbon.

[21] Williams G., Seger B., Kamat P.V. (2008). TiO_2_-graphene nanocomposites. UV-assisted photocatalytic reduction of graphene oxide. ACS Nano.

[22] Zhu Y., Murali S., Stoller M.D., Velamakanni A., Piner R.D., Ruoff R.S. (2010). Microwave assisted exfoliation and reduction of graphite oxide for ultracapacitors. Carbon.

[23] Kasischke M., Maragkaki S., Volz S., Ostendorf A., Gurevich E.L. (2018). Simultaneous nanopatterning and reduction of graphene oxide by femtosecond laser pulses. Appl. Surf. Sci..

[24] Baraket M., Walton S.G., Wei Z., Lock E.H., Robinson J.T., Sheehan P. (2010). Reduction of graphene oxide by electron beam generated plasmas produced in methane/argon mixtures. Carbon.

[25] Dey A., Krishnamurthy S., Bowen J., Nordlund D., Meyyappan M., Gandhiraman R.P. (2018). Plasma jet printing and in situ reduction of highly acidic graphene oxide. ACS Nano.

[26] Yang C.R., Tseng S.F., Chen Y.T. (2018). Laser-induced reduction of graphene oxide powders by high pulsed ultraviolet laser irradiations. Appl. Surf. Sci..

[27] Nguyen V.T., Le H.D., Nguyen V.C., Ngo T.T.T., Le D.Q., Nguyen X.N., Phan N.M. (2013). Synthesis of multi-layer graphene films on copper tape by atmospheric pressure chemical vapor deposition method. Adv. Nat. Sci. Nanosci. Nanotechnol..

[28] Gao X., Jang J., Nagase S. (2010). Hydrazine and thermal reduction of graphene oxide: reaction mechanisms, product structures, and reaction design. J. Phys. Chem. C.

